# Evaluation of Tuberculosis Preventive Treatment Uptake Among People Living with HIV in PEPFAR-Supported Facilities in Zimbabwe

**DOI:** 10.3390/tropicalmed10100296

**Published:** 2025-10-18

**Authors:** Collins Timire, Tawanda Mapuranga, Ronald T. Ncube, Talent Maphosa, Sithabiso Dube, Nqobile Mlilo, Cynthia Chiteve, Selma Dar Berger, Owen Mugurungi, Fungai Kavenga, Tsitsi Mutasa-Apollo, Manners Ncube, Clorata Gwanzura, Macarthur Charles, Riitta A. Dlodlo, Julia Ershova

**Affiliations:** 1 International Union Against Tuberculosis and Lung Disease (The Union), 75001 Paris, France; sberger@theunion.org (S.D.B.); rdlodlo@theunion.org (R.A.D.); 2 AIDS & TB Department, Ministry of Health and Child Care, Harare P.O. Box CY 1122, Zimbabwe; mugurungi@gmail.com (O.M.); drkav8@gmail.com (F.K.); tsitsiapollo1@gmail.com (T.M.-A.); ncube.manners@gmail.com (M.N.); cloratag@gmail.com (C.G.); 3 Union Zimbabwe Trust, 261 Diamond Road, Westgate, Harare P.O. Box CY 550, Zimbabwe; tmapuranga@uzt.org.zw (T.M.); rncube@uzt.org.zw (R.T.N.); sdube@uzt.org.zw (S.D.); nmlilo@uzt.org.zw (N.M.); cchiteve@uzt.org.zw (C.C.); 4 Department of Health and Human Services, U.S. Centers for Disease Control and Prevention, 1600 Clifton Road, Atlanta, GA 30329, USA; oxv9@cdc.gov (T.M.); xzk9@cdc.gov (M.C.); jhe3@cdc.gov (J.E.)

**Keywords:** isoniazid preventive therapy, TB infection, sub-Saharan Africa, childhood tuberculosis, collaborative TB-HIV services

## Abstract

Tuberculosis preventive treatment (TPT) reduces the incidence of tuberculosis (TB) among people living with HIV (PLHIV), but its coverage remains suboptimal in most settings. We conducted a cross-sectional study to describe TPT uptake among PLHIV and factors influencing TPT initiation. Healthcare workers (HCWs) in selected facilities were trained and supported to strengthen TPT management among PLHIV, including children living with HIV (CLHIV). Of 1309 enrolled PLHIV, 1268 (97%) were eligible for TPT; 1078 (85%) initiated TPT, including 663/776 (86%) among those currently on ART and 415/492 (84%) among clients newly on ART. The major reasons for not starting TPT included stock-outs of TPT medicines, TB disease, and refusal of TPT, mostly by CLHIV and adults currently on ART. Optimal and sustained uptake of TPT can be achieved through ensuring uninterrupted stocks of TPT medicines, including shorter regimens and pediatric formulations, addressing knowledge deficits among HCWs, and improving demand for TPT by educating PLHIV and caregivers of CLHIV about the benefits and risks of TPT formulations.

## 1. Introduction

A quarter of the global population is infected with *Mycobacterium tuberculosis* (*M. tb*) and have tuberculous (TB) infection [1]. If left untreated, infected persons have a 10–15% lifetime risk of developing TB disease [2,3]. The risk of progression from infection to TB disease is higher among people with a compromised immune system, such as people living with HIV (PLHIV) [3]. Antiretroviral therapy (ART) reduces the risk among PLHIV but needs to be complemented with TB preventive treatment (TPT) after TB has been ruled out [4].

The first pillar of the World Health Organization (WHO) End TB strategy, integrated patient centered care, aims to ensure early diagnosis and universal access to drug sensitivity testing (DST) using molecular WHO-recommended rapid diagnostics (WRDs) and to improve preventive treatment of persons at high risk of TB such as PLHIV and children (<5 years) who are contacts of people with TB [5,6]. However, focusing on the early diagnosis of TB may not be sufficient to halt community transmission. High-impact interventions such as TPT provision have the potential to be a game changer in global efforts to bend the epidemic curve towards eliminating TB by 2035 [5].

TPT helps to lessen reservoirs of TB, reducing the incidence of reactivation from TB infection and the development of TB disease [7]. Traditionally, isoniazid (H) taken daily for 6 (6H) or 9 (9H) months has been widely used among PLHIV. However, uptake and completion rates have been suboptimal, especially among children living with HIV (CLHIV), due to its long duration and associated side effects, such as peripheral neuropathy [8]. Shorter regimens such as 3HP (isoniazid plus rifapentine), taken orally as a once-weekly dose for three months, have proven to be as effective as 6H and are well tolerated with superior completion rates to 6H in various settings [6,9,10,11]. Despite the beneficial effects of TPT and the introduction of shorter and more tolerable and effective TPT regimens, coverage remains suboptimal in resource-limited settings [4]. Of the targeted 30 million PLHIV globally, only around 52% (15.5 million people) received TPT during the period 2018–2022 [12,13].

Zimbabwe has a high TB and HIV burden, with a co-infection rate of 51%, and an HIV-positive TB incidence of 125 per 100,000 population [12,14]. The country remains on the global list of high-burden countries for HIV-associated TB as well as for multidrug-resistant and rifampicin-resistant (MDR/RR) TB. According to a patient cost survey conducted in the country in 2018, 80% of TB patients in Zimbabwe faced catastrophic expenditures and 26% were expected to die due to TB; this underscores an urgent need to reduce TB morbidity and mortality in the country [15]. To follow the End TB strategy recommendation on implementing TPT for populations at high risk of developing TB, including PLHIV, the Zimbabwe Ministry of Health and Child Care began rolling out TPT to reduce TB burden among PLHIV in 2012. The United States Centers for Disease Control and Prevention (CDC) through the U.S. President’s Emergency Plan for AIDS Relief (PEPFAR) has been supporting TPT implementation in Zimbabwe since 2019. TPT coverage among PLHIV in Zimbabwe was suboptimal at 39% and 17% in 2020 and 2021, respectively [16]. In 2022, CDC PEPFAR provided additional support aimed at strengthening the supply of TPT. The support also included training of HCWs to strengthen diagnostic capacity to rule out TB disease and prescribe TPT, including shorter regimens [16].

We conducted a study to describe (i) TPT uptake among PLHIV who newly initiated ART, including CLHIV, and those currently on ART at the start of the study, before and during the study period; (ii) factors associated with not starting TPT; and (iii) adverse events associated with TPT implementation.

## 2. Methods

We used a prospective study design to enroll PLHIV in care until the target sample size at each facility was reached. The study was conducted in 12 PEPFAR-supported facilities in three provinces of Zimbabwe: Mashonaland East, Central, and West. The facilities were selected based on high TB and HIV burden and low uptake of TPT. The selected facilities reflected a mix of rural/urban clinics, district hospitals and provincial hospitals, as well as facilities that utilize both paper-based and electronic health records. During enrollment within a facility, all available documentation, including medical charts from eligible adult and pediatric patients, as well as ART, TPT, and TB Registers, were used for data abstraction.

All PLHIV visiting the selected health facilities from 1 August 2022, until the study sample size was reached were eligible for evaluation irrespective of TPT or TB status. PLHIV who started ART prior to 1 August 2022 were considered currently on ART, while those who started ART on or after 1 August 2022 were considered newly on ART. These two categories were further classified into adults (≥15 years) and children (<15 years), thus resulting in four categories: adults currently on ART (AC), adults newly on ART (AN), children currently on ART (CC), and children newly on ART (CN).

We used existing TB/TPT programmatic data available for PLHIV and CLHIV in Zimbabwe and a design effect of two to estimate the number of clients needed for an assessment of TPT initiation for each of the four PLHIV cohorts in our study. Overall, a sample size of 1360 PLHIV was calculated to ensure the generalizability of the results, including 500 adult PLHIV per cohort (current and new on ART); 300 children currently on ART; and 60 children new on ART. Each facility was allocated a sample size proportional to size, proxied by case load of PLHIV on ART. Facility-specific sample sizes were disaggregated by age group and enrollment category.

The evaluation was embedded within a routine program setting. Prior to the implementation, health managers and HCWs were trained on protocol procedures; refresher training on TPT initiation, including new TPT regimens (3HP and 3HR), TPT monitoring, and recording, was conducted by the study team. Each enrolled participant was assigned a unique identification number which was entered in the Enrollment Register. The register also included the date of enrollment and demographic details such as age, sex, ART cohort number, address, and name for tracking purposes. This information was stored at the facility in locked cabinets and was only used by the study team during data verification.

### 2.1. Screening for TB Symptoms

At each visit to the study health facility, PLHIV were routinely screened for TB symptoms using the four-symptom screen tool (W4SS) (as per national TB screening guidelines) and referred for chest X-ray (CXR) where available [17]. Clients with TB symptoms were asked to submit spot sputum specimens for Xpert MTB/RIF (Cepheid, Sunnyvale, CA, USA) or Truenat (Molbio Diagnostics, Verna, Goa, India). In case the necessary laboratory services were unavailable at the facility, a sputum transportation system was in place to transport specimens to a nearby designated laboratory.

### 2.2. TPT Eligibility Assessments, Initiations and Monitoring

PLHIV who screened negative for TB symptoms, had a CXR with no radiologic signs of TB, or received a negative Xpert MTB/RIF test result were assessed for TPT eligibility. Those who had not taken TPT within the last three years and had no contraindications (those with TB disease, heavy alcohol users, clients with acute or chronic liver disease, those taking drugs that may potentially interact with the TPT regimen, those undergoing TB treatment, and those with allergies to any drugs in the TPT regimen) were considered eligible and were offered TPT per the national TB/HIV management guidelines [17,18]. The dispensing of TPT was synchronized with ART refills. TPT-related information (TB screening results, TPT eligibility, contraindications, and adverse events) was collected and documented in paper-based patient ART booklets and the TPT Register by HCWs during each routine ART visit. A mechanism for verifying adherence to the TPT regimen was not established in the country. TPT outcomes were self-reported by PLHIV during ART refill visits or in response to calls made by TB nurses to inquire about TPT completion.

Support and mentorship visits were conducted by the study team quarterly to ensure progress and verify enrollments, check stocks of both TPT and ancillary medicines, and review ART registers for any PLHIV who may have been missed during enrollment.

### 2.3. Data Collection

The study team visited selected health facilities for data abstraction three times during the enrollment period. Data from the enrolled PLHIV were abstracted in a standardized paper-based form that included patient study ID, information on the enrollment dates, enrollment category, demographic information, ART initiation, and TB and TPT status prior to and during enrollment, including any adverse events related to prior and current TPT uptake. A separate form was used if the patient had any form of TB to capture data on diagnosis, treatment, and care for TB. Some of the data on TPT status (especially for PLHIV who had been on ART for ≥5 years prior to enrollment in the project) were not available in paper-based registers and had to be obtained from the electronic patient management system. The enrollment register included an option to indicate whether data abstraction for each specific patient was carried out on each visit. Epi Data (EpiData Association, Odense, Denmark) version 4.6.0.6 was used for electronic data collection for the project [19].

### 2.4. Data Analysis

Data were analyzed in Epi Info version 7.2.6.0 and EpiData version 2.2.3.187 (EpiData Association, Odense, Denmark) [19,20]. Demographic and clinical data were analyzed using descriptive statistics: categorical variables using numbers and proportions and continuous variables using means and standard deviations, as appropriate. The key outcome variable for this analysis was TPT initiation at the last ART visit before and during the study period. It was a binary variable indicating whether TPT was started (Yes/No). Logistic regression was conducted to assess factors associated with TPT initiation before and after the start of the project.

### 2.5. Ethics

Ethics approval was obtained from the Medical Research Council of Zimbabwe (MRCZ/A/2746). This project was reviewed by CDC, was deemed not research, and was conducted in a manner consistent with applicable federal law and CDC policy.

## 3. Results

We enrolled 1309 (96%) PLHIV of the targeted 1360 sample size. Of these, 758 (58%) were female, 970 (74%) were adults (≥15 years), and 530 (40%) were newly on ART (Table 1). The median ages for PLHIV newly on ART and currently on ART were 34 (IQR = 15–44) years and 31 (IQR = 12–45) years, respectively.

### 3.1. TPT Initiation Among PLHIV

**Figure 1:**
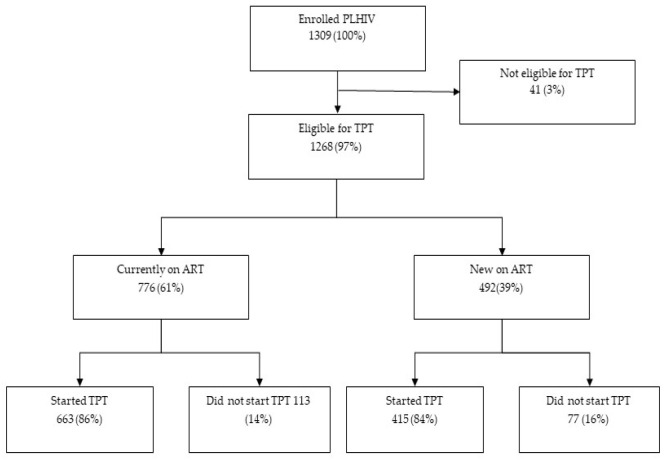
TPT initiations among PLHIV enrolled in the study. PLHIV = People living with HIV; ART = Antiretroviral therapy; TPT = Tuberculosis preventive treatment.

Of the 1309 PLHIV enrolled in the study, 1268 (97%) were eligible for TPT. Among 41 non-eligible for TPT, 38 had TB disease and 3 were heavy alcohol users. Overall, 1078/1268 (85%) initiated TPT, including 663/776 (86%) among those currently on ART and 415/492 (84%) among clients newly on ART (Figure 1). Information on the TPT regimen was available for 712/1078 (66%) PLHIV; most clients (519/712, 73%) initiated the 3HP regimen, whereas 193/712 (27%) initiated the 6H regimen.

### 3.2. Reasons for Not Starting TPT

Among 1268 eligible PLHIV, 190 (15%) did not start TPT. The reasons for not starting TPT were available for 137/190 (72%) clients; this information was missing for 53 (28%) clients (Table 2). Among adults currently on ART (AC) who did not start TPT, 53% (24/45) refused it. Stock-out of TPT medicines was the most frequent reason among 97.5% (38/39) of children currently on ART (CC) and among 100% (15/15) of children newly on ART (CN).

### 3.3. Adverse Events of TPT

Adverse events were reported by six (0.5%) clients, including three children. None of the reported events were life-threatening. Three of six clients (including one child) reported peripheral neuropathy. TPT was stopped due to this adverse event in all three clients. Three other clients reported minor adverse events due to isoniazid (H), including skin rash; TPT was not interrupted in these clients.

### 3.4. Factors Associated with Not Initiating TPT Before the Start of the Study

Of the 776 PLHIV who were currently on ART at the start of the study, 663 (85%) initiated TPT, including 337 initiating TPT before the study started. The association between TPT initiation and sociodemographic and clinical characteristics before the study period is shown in Table 3. Men had 1.5 times higher odds of not starting TPT than women. The odds of not starting TPT were higher among children <5 years (OR = 3.39 (95% CI: 1.11–10.39)) and PLHIV within the age category 35–44 years (OR = 1.78 (95% CI: 1.03–3.08)) compared to the age group of 25–34 years old, as well as among PLHIV from Mashonaland West and East provinces compared to those frpm Mashonaland Central province (OR = 15.69 (95% CI: 8.75–28.11) and OR = 19.48 (95% CI: 10.79–35.15), respectively).

### 3.5. Factors Associated with Not Initiating TPT During the Study Period

During the study period, 326 clients who were currently on ART and 415 of those newly on ART started TPT. Dates from 37 clients were not available to ensure the proper classification of patients; hence, the records were excluded from the analytical dataset. The association between TPT initiation and patient characteristics during the study period is shown in Table 4 below.

During the study period, the odds of not starting TPT were higher among children (<15 years) compared to adults, including among those <5 years old (OR = 22.05 (95% CI: 8.21–59.21)), among those 5–14 years old (OR = 3.60 (95% CI: 1.87–6.93)), and among PLHIV in Mashonaland West province compared to Mashonaland Central (OR = 2.88 (95% CI: 1.51–5.47)). Adults newly on ART were more likely to initiate TPT compared to adults that were on ART before the study started: OR = 0.41 (95% CI: 0.26–0.66). Children were less likely to initiate TPT compared to the same category of adults, including those newly on ART CLHIV (OR = 5.02 (95% CI: 2.23–11.30)) and CLHIV already on ART at the start of the study (OR = 1.83 (95% CI: 1.11–2.99)).

### 3.6. TPT Initiations Recorded During the Follow-Up Visits

Overall, 41 clients initiated TPT between their enrollment in the study and the follow-up visits of the study team for data abstraction. This includes 22 clients who initiated TPT by the first follow-up visit and another 19 clients who started TPT by the second visit. Among 41 clients were 9 adults on ART at the start of the study (AC) who had TB disease at enrollment; they initiated TPT when TB treatment was completed during the study period. Seven AC clients who previously refused to initiate TPT also started it later during their follow-up ART visits. Finally, 22 clients who could not initiate TPT due to stock-out of medicine at the enrollment were also able to initiate TPT later during the study. Information from the other three clients who initiated TPT later after the enrollment was missing.

## 4. Discussion

In an effort to strengthen TPT among PLHIV in Zimbabwe, we aimed to determine the coverage and adverse events for TPT in PEPFAR-supported facilities. We observed a high uptake of TPT during the study period and a very low proportion of adverse events due to TPT. TPT uptake differed among different PLHIV categories and was lowest among new CLHIV.

Prior to the study period, TPT uptake was comparable to uptakes reported in several settings [12,13]. Six months of isoniazid was the most frequently used TPT regimen prior to the study period, and several barriers to its uptake (e.g., side effects, long duration) have been reported among adults and adolescents [21,22]. More than 70% of PLHIV enrolled in our study received 3HP, a shorter regimen with a high acceptance among them [9]. As a result, TPT uptake during the study period was higher than the global average of 56% [12] and comparable to results from South Africa [2,23]. However, the high acceptance of shorter TPT regimens does not always translate into high uptake of TPT in program settings, highlighting the existence of contextual barriers to TPT uptake [13,21].

Health system-related factors such as stock-outs of TPT and related medicines, including pyridoxine, have been reported during our study, resulting in essentially missed opportunities for TPT uptake. Similar factors were described in Nigeria, India, and Uganda [24,25,26,27]. Moreover, PLHIV who were currently on ART and previously did not complete TPT due to stock-outs of TPT refused TPT, probably because TPT interruptions altered their health-seeking behavior. Effective TPT implementation requires sustained and uninterrupted stocks of TPT medicines, especially shorter regimens.

Further, PLHIV often lack adequate knowledge about TPT and view HCWs as agents who determine their treatment plans. Relationships and effective communication between PLHIV and HCWs are therefore crucial to improving TPT uptake and adherence, bringing to the fore the unique role HCWs can play as either facilitators or barriers to TPT uptake.

In this study, some HCWs expressed hesitancy to start CLHIV under 5 years of age on TPT. HCWs who lacked adequate knowledge about TPT may have given unclear or unsatisfactory explanations to parents and/or caregivers on the importance of TPT [28]. The uptake of TPT among CLHIV could improve if shorter and more palatable TPT regimens were available to this group. Cruz and Starke observed that children and adolescents (0–18 years) have better tolerance and adherence to 3HP than 9H [22]. The low uptake of TPT among CLHIV in this study can be attributed to the use of 6H owing to the lack of guidance (during the study period) on use on shorter regimens among this group.

PLHIV usually hesitate to take TPT, citing a fear of adverse effects [2,13]. This study has revealed a very low (<1%) proportion of adverse effects, none of which were life-threatening. The proper quantification of adverse events due to TPT is crucial, as it can allay fears about TPT among PLHIV and caregivers of CLHIV.

The study was conducted in a program setting and our results reflect a real-world situation. We unmasked the differential uptake of TPT by disaggregating TPT uptake among four categories of PLHIV. This may inform focused interventions to improve TPT uptake among CLHIV and adult PLHIV in care. The study took place in the PEPFAR-supported facilities where staff received appropriate training on TPT and additional support for the utilization of shorter TPT regimens; thus, our results may not be generalizable for the whole of Zimbabwe. However, PEPFAR support is provided to the majority of HIV facilities in Zimbabwe, and we believe that our results represent the majority of HIV services in the country. We did not include data for approximately 50 PLHIV in our analytic dataset since we did not have sufficient data to establish whether they received TPT prior to or during the study period. The program transitioned from old to new paper-based patient monitoring booklets across the country, and key TPT events were recorded as a carryover summary. Some data that were key for our study, e.g., dates, were not captured consistently. Lastly, we lacked data on the socioeconomic and education status of parents/caregivers of CLHIV. These variables may invariably affect both TPT uptake and adherence among CLHIV. However, since TB disproportionally affects people who are on the lower rungs of the social ladder, we reckon that there is little heterogeneity within our study population with respect to these variables. Hence, the associations we observed here may not change.

Despite these limitations, our study highlights several demand and supply factors that can be addressed to ensure optimal TPT uptake. First, optimal uptake of TPT requires uninterrupted supplies of TPT, especially shorter regimens. This can be ensured through effective communication between pharmacies, clinical departments, and the national TB program to ensure adequate stocks are maintained. Second, this study has highlighted the differential uptake of TPT, with the lowest uptake among CLHIV. Introducing innovative ways to improve the uptake of TPT among children and adults who have been on ART for a long time could be beneficial. The provision of shorter regimens alone may not be the solution. It is important to take into account the preferences of CLHIV regarding the taste of TPT medicines and the timely delivery of health services [29]. Third, creating demand for TPT through the provision of information, education, and communication (IEC) materials to caregivers of CLHIV and adult PLHIV could help to improve TPT uptake among HIV-affected populations. This can be supplemented with community awareness campaigns aimed at educating people on the benefits and risks of TPT and dispelling misconceptions about TPT. Community health workers may be tasked to ask for slots to deliver health talks during community meetings. Fourth, against the backdrop that some PLHIV throw away TPT medicines and that adherence to TPT was based on pill counts and self-reports by PLHIV, future studies can harness digital adherence technologies such as smart pill boxes and video-supported treatment to ensure that once TPT is dispensed to PLHIV, it is actually taken [30]. Lastly, prioritizing comprehensive TPT training for a sufficient number of HCWs in charge of TB/HIV clinics could ensure better outcomes. The training may focus on optimizing the staff attitude during interface with PLHIV as well as knowledge and skills about TPT.

## 5. Conclusions

Optimal and sustained TPT uptake requires context-specific interventions addressing demand and supply for TPT. Demand for TPT can be improved by addressing knowledge deficits among PLHIV, caregivers of CLHIV, and people within communities. Supply factors can be improved by capacitating HCWs with adequate knowledge about TPT and ensuring uninterrupted stocks of TPT, especially shorter regimens.

## Figures and Tables

**Table 1:** Sociodemographic and clinical characteristics of PLHIV enrolled in the study, n = 1309.

		Total		Newly on ART		Currently on ART	
Characteristic		N	(%)	n	(%)	n	(%)
		1309	(100)	530	(100)	779	(100)
Sex	Male	549	(42)	230	(43)	319	(41)
	Female	758	(58)	300	(57)	458	(59)
	Not recorded	2	(<1)	-	-	2	(<1)
Age category	<5	52	(4)	26	(5)	26	(3)
	5–14	287	(22)	20	(4)	84	(11)
	15–24	131	(10)	86	(16)	228	(28)
	25–34	229	(18)	140	(26)	89	(11)
	35–44	314	(24)	165	(31)	149	(19)
	45+	292	(22)	91	(17)	201	(26)
	Not recorded	4	(<1)	2	(<1)	2	(<1)
Enrollment category	Adults (≥15 years)	970	(74)	484	(91)	486	(62)
	Children (<15 years)	339	(26)	46	(9)	293	(38)
Province	Mashonaland West	531	(41)	219	(41)	312	(40)
	Mashonaland East	465	(35)	179	(34)	286	(37)
	Mashonaland Central	313	(24)	132	(25)	181	(23)
Health facility	Chinhoyi hospital	163	(13)	45	(8)	118	(15)
	Marondera hospital	162	(12)	48	(9)	114	(15)
	Concession hospital	142	(11)	71	(13)	71	(9)
	Katanga Utano clinic	111	(9)	60	(11)	51	(7)
	Ruwa hospital	110	(8)	40	(8)	70	(9)
	Dombotombo clinic	104	(8)	48	(9)	56	(7)
	Chikonohono clinic	93	(7)	51	(10)	42	(5)
	Howard hospital	92	(7)	28	(5)	64	(8)
	Macheke clinic	89	(7)	43	(8)	46	(6)
	Kuwadzana clinic	85	(7)	40	(8)	45	(6)
	Raffingora clinic	79	(6)	23	(4)	56	(7)
	Ruyamuro clinic	79	(6)	33	(6)	46	(6)

**Table 2:** Reasons for not starting TPT among those eligible, by enrollment category, n = 137.

Reasons for Not Starting TPT	Adult Currently on ART n = 45	Adult Newly on ART n = 38	Child Currently on ART n = 39	Child Newly on ART n = 15
Stock-out of TPT medicine	21 (47%)	36 (95%)	38 (97.5%)	15 (100%)
Refusal	24 (53%)	2 (5%)	1 (2.5%)	0 (0%)

**Table 3:** Factors associated with not starting TPT before the start of the study (n = 710).

			Started TPT				
PLHIV		Total	No		Yes		OR (95% CI)
			n	(%) *	n	(%) *	
All		710	373	(53)	337	(47)	
Sex	Male	292	170	(58)	122	(42)	1.49 (1.10–2.02)
	Female	416	201	(48)	215	(52)	Ref
Age category (years)	<5	18	13	(57)	5	(43)	3.39 (1.11–10.39)
	5–14	238	119	(50)	119	(50)	1.31 (0.79–2.16)
	15–24	41	23	(56)	18	(44)	1.67 (0.78–3.55)
	25–34	83	36	(43)	47	(57)	Ref
	35–44	142	82	(58)	60	(42)	1.78 (1.03–3.08)
	45+	188	100	(53)	88	(47)	1.48 (0.88–2.49)
Province	Mashonaland West	287	179	(62)	108	(38)	15.69 (8.75–28.11)
	Mashonaland East	266	179	(67)	87	(33)	19.48 (10.79–35.15)
	Mashonaland Central	157	15	(10)	142	(90)	Ref
Enrollment category	CC	256	124	(48)	132	(52)	0.94 (0.69–1.28)
	AC	454	241	(53)	213	(47)	Ref

* = Row percentages; OR = Odds ratio; CI = Confidence interval; TPT = TB preventive treatment.

**Table 4:** Factors associated with not starting TPT among PLHIV during the study period (n = 877).

			Started TPT				
PLHIV		Total	No		Yes		OR (95% CI)
			n	(%) *	n	(%) *	
All		841	137	(16)	704	(84)	
Sex	Male	367	56	(15)	311	(85)	1.05 (0.75–1.46)
	Female	472	80	(17)	392	(83)	Ref
Age category (years)	<5	26	18	(70)	8	(30)	22.05 (8.21–59.21)
	5–14	134	36	(27)	98	(73)	3.60 (1.87–6.93)
	15–24	105	17	(16)	88	(84)	1.89 (0.90–3.97)
	25–34	162	15	(9)	147	(91)	Ref
	35–44	233	25	(11)	208	(89)	1.18 (0.60–2.31)
	45+	181	26	(14)	155	(86)	1.64 (0.83–3.23)
Province	Mashonaland West	373	84	(23)	289	(77)	2.88 (1.51–5.47)
	Mashonaland East	337	41	(12)	296	(88)	1.37 (0.69–2.70)
	Mashonaland Central	131	12	(9)	119	(91)	Ref
Enrollment category	CN	28	15	(54)	13	(46)	5.02 (2.23–11.30)
	CC	132	39	(30)	93	(70)	1.83 (1.11–2.99)
	AN	440	38	(9)	402	(91)	0.41 (0.26–0.66)
	AC	241	45	(19)	196	(81)	Ref

* = Row percentages; OR = Odds ratio; CI = Confidence interval; TPT = TB preventive treatment; CC = Children currently on ART; CN = children newly on ART; AN = Adults newly on ART; AC = Adults currently on ART.

## Data Availability

The data presented in this study are available on request from the corresponding author. The data are not publicly available due to authorizations that may be required by the funder.

## References

[1] Houben R.M.G.J., Dodd P.J. (2016). The Global Burden of Latent Tuberculosis Infection: A Re-estimation Using Mathematical Modelling. PLoS Med..

[2] Nyarubamba R.F., Silumbwe A., Jacobs C., Maritim P., Mdoe P., Zulu J.M. (2022). Assessment of contextual factors shaping delivery and uptake of isoniazid preventive therapy among people living with HIV in Dar es salaam, Tanzania. BMC Infect. Dis..

[3] World Health Organisation (2018). Latent Tuberculosis Infection: Updated and Consolidated Guidelines for Programmatic Management.

[4] Pathmanathan I., Ahmedov S., Pevzner E., Anyalechi G., Modi S., Kirking H., Cavanaugh J.S. (2018). TB preventive therapy for people living with HIV: Key considerations for scale-up in resource-limited settings. Int. J. Tuberc. Lung Dis..

[5] STOP TB Partnership (2015). The Paradigm Shift 2016–2020: Global Plan to End TB.

[6] STOP TB Partnership (2019). The Paradigm Shift, Global Plan to End TB: 2018–2022.

[7] World Health Organisation (2020). WHO Consolidated Guidelines on Tuberculosis. Module 1: Prevention—Tuberculosis Preventive Treatment.

[8] Shivaramakrishna H.R., Frederick A., Shazia A., Murali L., Satyanarayana S., Nair S.A., Kumar A.M., Moonan P.K. (2014). Isoniazid preventive treatment in children in two districts of South India: Does practice follow policy?. Int. J. Tuberc. Lung Dis..

[9] Semitala F.C., Kadota J.L., Musinguzi A., Nabunje J., Welishe F., Nakitende A., Akello L., Bishop O., Patel D., Sammann A. (2021). Completion of isoniazid–rifapentine (3HP) for tuberculosis prevention among people living with HIV: Interim analysis of a hybrid type 3 effectiveness–implementation randomized trial. PLoS Med..

[10] Borse R., Randive B., Mattoo S., Malik P., Solanki H., Gupta A., Chaisson R., Mave V., Suryavanshi N. (2024). Three months of weekly rifapentine plus isoniazid for TB prevention among people with HIV. IJTLD Open.

[11] Rahman T., Hossain F., Banu R.S., Islam S., Alam S., Faisel A.J., Salim H., Cordon O., Suarez P., Hussain H. (2023). Uptake and Completion of Tuberculosis Preventive Treatment Using 12-Dose, Weekly Isoniazid–Rifapentine Regimen in Bangladesh: A Community-Based Implementation Study. Trop. Med. Infect. Dis..

[12] World Health Organisation (2024). Global TB Report 2024.

[13] Nyathi S., Dlodlo R.A., Satyanarayana S., Takarinda K.C., Tweya H., Hove S., Matambo R., Mandewo W., Nyathi K., Sibanda E. (2019). Isoniazid preventive therapy: Uptake, incidence of tuberculosis and survival among people living with HIV in Bulawayo, Zimbabwe. PLoS ONE.

[14] (2024). Country Factsheets: Zimbabwe UNAIDS. https://www.unaids.org/en/regionscountries/countries/zimbabwe.

[15] Timire C., Ngwenya M., Chirenda J., Metcalfe J.Z., Kranzer K., Pedrazzoli D., Takarinda K.C., Nguhiu P., Madzingaidzo G., Ndlovu K. (2021). Catastrophic costs among tuberculosis-affected households in Zimbabwe: A national health facility-based survey. Trop. Med. Int. Health.

[16] World Health Organisation Zimbabwe Strengthens Capacity to Increase Tuberculosis Preventive Therapy Coverage. https://www.afro.who.int/countries/zimbabwe/news/zimbabwe-strengthens-capacity-increase-tuberculosis-preventive-therapy-coverage#:~:text=Zimbabwe%20has%20experienced%20a%20decrease,quickly%20commence%20them%20on%20TPT.

[17] Ministry of Health and Child Care (2020). Management of Latent Tuberculosis Infection Among People Living with HIV and Tuberculosis Contacts: An Addendum to the 2016 Guidelines for Anti-retroviral Therapy for Prevention and Treatment of HIV and Tuberculosis and Leprosy Management in Zimbabwe.

[18] Sandy C., Takarinda K.C., Timire C., Mutunzi H., Dube M., Dlodlo R.A., Harries A.D. (2020). Preparing national tuberculosis control programmes for COVID-19. Int. J. Tuberc. Lung Dis..

[19] Epi Data Software. https://www.epidata.dk/downloads/epidataflyer_general.pdf.

[20] Epi Info Software. https://www.cdc.gov/epiinfo/support/downloads.html.

[21] Sharma N., Basu S., Khanna A., Sharma P., Chopra K.K., Chandra S. (2022). Adherence to Isoniazid Preventive Therapy among children living with tuberculosis patients in Delhi, India: An exploratory prospective study. Indian J. Tuberc..

[22] Cruz A.T., Starke J.R. (2018). Completion Rate and Safety of Tuberculosis Infection Treatment with Shorter Regimens. Pediatrics.

[23] Johnson A., Chimoyi L., Charalambous S., Kawaza N., Hoffmann C.J., Davis J.L., Chihota V. (2025). Differentiated HIV Service Delivery vs Conventional Care: Tuberculosis Preventive Therapy Outcomes for People Living with HIV in Sub-Saharan Africa. MedRxiv.

[24] Lester R., Hamilton R., Charalambous S., Dwadwa T., Chandler C., Churchyard G.J., Grant A.D. (2010). Barriers to implementation of isoniazid preventive therapy in HIV clinics: A qualitative study. AIDS.

[25] Ihesie A., Chukwuogo O., Eneogu R., Daniel O.K., Agbaje A., Odume B., Nongo D., Ohikhuai C., Kadiri-Eneh N., Oyelaran O. (2024). Acceptance and Completion Rates of 3-Month Isoniazid-Rifampicin (3HR) Tuberculosis Preventive Treatment (TPT) Among Contacts of Bacteriologically Confirmed TB Patients—Patients’ and Healthcare Workers’ Perspectives. Trop. Med. Infect. Dis..

[26] Singh A.R., Kharate A., Bhat P., Kokane A.M., Bali S., Sahu S., Verma M., Nagar M., Kumar A.M. (2017). Isoniazid Preventive Therapy among Children Living with Tuberculosis Patients: Is It Working? A Mixed-Method Study from Bhopal, India. J. Trop. Pediatr..

[27] Eurien D., Okethwangu D., Aliddeki D.M., Kisaakye E., Nguna J., Bulage L., Mugerwa S., Ario A.R. (2024). Low completion rate for the 6-months course of isoniazid preventive therapy among people living with HIV, North Eastern Uganda, 2015–2017. Pan Afr. Med. J..

[28] An Y., Teo A.K.J., Huot C.Y., Tieng S., Khun K.E., Pheng S.H., Leng C., Deng S., Song N., Nonaka D. (2023). They do not have symptoms—Why do they need to take medicines? Challenges in tuberculosis preventive treatment among children in Cambodia: A qualitative study. BMC Pulm. Med..

[29] Strauss M., Wademan D.T., Mcinziba A., Hoddinott G., Rafique M., Jola L.N., Streicher C., du Preez K., Osman M., Boffa J. (2023). TB preventive therapy preferences among children and adolescents. Int. J. Tuberc. Lung Dis..

[30] STOP TB Partnership Digital Adherence Technologies. https://www.stoptb.org/what-we-do/accelerate-tb-innovations/digital-health-technology-hub/digital-adherence-technologies.

